# Causal Inference in Multisensory Perception

**DOI:** 10.1371/journal.pone.0000943

**Published:** 2007-09-26

**Authors:** Konrad P. Körding, Ulrik Beierholm, Wei Ji Ma, Steven Quartz, Joshua B. Tenenbaum, Ladan Shams

**Affiliations:** 1 Rehabilitation Institute of Chicago, Northwestern University, Chicago, Illinois, United States of America; 2 Computation and Neural Systems, California Institute of Technology, Pasadena, California, United States of America; 3 Department of Brain and Cognitive Sciences, University of Rochester, Rochester, New York, United States of America; 4 Division of Humanities and Social Sciences, California Institute of Technology, Pasadena, California, United States of America; 5 Department of Brain and Cognitive Sciences, Massachusetts Institute of Technology, Cambridge, Massachusetts, United States of America; 6 Department of Psychology, University of California at Los Angeles, Los Angeles, California, United States of America; Indiana University, United States of America

## Abstract

Perceptual events derive their significance to an animal from their meaning about the world, that is from the information they carry about their causes. The brain should thus be able to efficiently infer the causes underlying our sensory events. Here we use multisensory cue combination to study causal inference in perception. We formulate an ideal-observer model that infers whether two sensory cues originate from the same location and that also estimates their location(s). This model accurately predicts the nonlinear integration of cues by human subjects in two auditory-visual localization tasks. The results show that indeed humans can efficiently infer the causal structure as well as the location of causes. By combining insights from the study of causal inference with the ideal-observer approach to sensory cue combination, we show that the capacity to infer causal structure is not limited to conscious, high-level cognition; it is also performed continually and effortlessly in perception.

## Introduction

Imagine you are walking in the forest and you see a sudden movement in the bushes. You may infer that this movement was caused by a hidden animal, but you may also consider a gust of wind as an alternative and possibly more probable cause. If you are a hungry predator–or a life-loving prey–this estimation may be critical to your survival. However, you may also hear an animal vocalization coming from a similar direction. Combining both pieces of sensory information, you will be better at judging if there is an animal in the bushes and if so, where exactly it is hiding. Importantly, the way how you will combine pieces of information must depend on the causal relationships you inferred. This example illustrates that perceptual cues are seldom ecologically relevant by themselves, but rather acquire their significance through their meaning about their causes. It also illustrates how cues from multiple sensory modalities can be used to infer underlying causes. The nervous system is constantly engaged in combining uncertain information from different sensory modalities into an integrated understanding of the causes of sensory stimulation.

The study of multisensory integration has a long and fruitful history in experimental psychology, neurophysiology, and psychophysics. Von Helmholtz, in the late 19^th^ century started considering cue combination, formalizing perception as unconscious probabilistic inference of a best guess of the state of the world [1]. Since then, numerous studies have analyzed the way people use and combine cues for perception [e.g. 2], [3], highlighting the rich set of effects that occur in multimodal perception.

Over the last decade, many scientists have gone back to a probabilistic interpretation of cue combination as had been proposed by von Helmholtz. These probabilistic models formalize the problem of cue combination in an elegant way. It is assumed that there is a single variable in the outside world (e.g., the position of an animal) that is causing the cues (auditory and visual information). Each of the cues is assumed to be a noisy observation of this underlying variable. Due to noise in sensation, there is some uncertainty about the information conveyed by each cue and Bayesian statistics is the systematic way of predicting how subjects could optimally infer the underlying variables from the cues. Several recent studies have demonstrated impressive fits to psychophysical data, starting from the assumption that human performance is close to the ideal defined by probabilistic models [4]–[7]. In these experiments, cues tend to be close to each other in time, space, and structure, providing strong evidence for there only being a single cause for both cues. In situations where there is only a single underlying cause, these models formalize the central idea of probabilistic inference of a hidden cause.

A range of experiments have shown effects that are hard to reconcile with the single-cause (i.e., forced-fusion) idea. Auditory-visual integration breaks down when the difference between the presentation of the visual and the auditory stimulus is large [8]–[10]. Such a distance or inconsistency is called disparity. Increasing disparity, for example by moving an auditory stimulus farther away from the position of a visual stimulus, reduces the influence each stimulus has on the perception of the other [11]–[14]. Throughout this paper we only consider spatial disparity along the azimuthal axis. When subjects are asked to report their percepts in both modalities *on the same trial*, one can measure the influence that the two senses have on each other [13]. The data from such a dual-report paradigm show that, although at small disparities there is a tendency to integrate, greater disparity makes it more likely that a subject responds differently in both modalities. Moreover, when people are simply asked whether they perceive a single cue or several cues they give answers that intuitively make a lot of sense: if two events are close to each other in space, time, and structure, subjects tend to perceive a single underlying cause, while if they are far away from one another subjects tend to infer two independent causes [15], [16]. If cues are close to one another, they interact and influence the perception of each other, whereas they are processed independently when the discrepancy is large.

New modeling efforts have made significant progress at formalizing the interactions between two cues. These models assume that there exist *two* relevant variables in the world, for example the position of a visual and the position of an auditory stimulus. The visual and auditory cues that reach the nervous system are noisy versions of the underlying visual and auditory variables. The models further assume an “interaction prior”, a joint prior distribution that defines how likely each combination of visual and auditory stimuli is in the absence of any evidence. This prior formalizes that the probability of both positions being the same (related to a common cause) is high in comparison to the positions being different from one another. This prior in effect determines the way in which two modalities influence each other. Very good fits to human performance have been shown for the combination of two cues [13], [14], [17], [18]. These studies assume an interaction between processing in each modality and derive predictions of human performance from this idea.

Von Helmholtz did not only stress the issue of probabilistic inference but also that multiple objects may be the causes of our sensations [19], [20]. In other words, any two sensory signals may either have a common cause, in which case they should be integrated, or have different, independent causes, in which case they should be processed separately. Further evidence for this idea comes from a study that showed that by providing evidence that two signals are related it is possible to incite subjects to more strongly combine two cues [21]. The within-modality binding problem is another example where causal inference is necessary and the nervous system has to determine which set of stimuli correspond to the same object and should be bound together [22]–[24]. We are usually surrounded by many sights, sounds, odors, and tactile stimuli, and our nervous system constantly needs to estimate which signals have a common cause. The nervous system frequently needs to solve problems where it needs to interpret sensory signals in terms of potential causes.

In this paper we formalize the problem of causal inference as well as integration versus segregation in multisensory perception as an optimal Bayesian observer that not only infers source location from two sensory signals (visual, s*_V_*, and auditory, s*_A_*) but also whether the signals have a common cause (*C*). This inference is complicated by the fact that the nervous system does not have access to the source locations of the signals but only to noisy measurements thereof (visual, *x_V_*, and auditory, *x_A_*). From these noisy observations it needs to infer the best estimates of the source locations (*Ŝ_V_* and *Ŝ_A_*). All this needs to happen in the presence of uncertainnty about the presence of a common cause (*C*). To take into account multiple possible causal structures, we need a so-called mixture model [e.g. 24], but one of a very specific form.

The model assumes that the underlying variables (azimuthal stimulus positions) cause the sensory inputs. The model considers two hypotheses, either that there is a common cause or that there are independent causes. The optimal observer model defines how the cues might actually be combined (i.e., in a statistically optimal manner). In the model, cues are fused if the cues have one common cause and segregated if they have independent causes. The model typically has uncertainty about the causal interpretation, in which case it will adjust its cue combination continuously depending on the degree of belief about the causal structure.

This model makes three important predictions: (1) It predicts the circumstances under which subjects should perceive a common cause or independent causes. (2) It predicts if the individual cues should be fused or if they should be processed separately. (3) It predicts how the cues are combined if they are combined. Here we test the predictions of the model and analyze how well it predicts human behavior.

## Results

### Causal Bayesian inference

**Figure 1:**
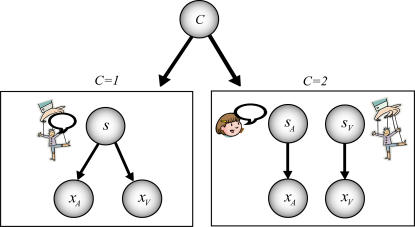
The causal inference model. Left: One cause can be responsible for both cues. In this case the visually perceived position *x_V_* will be the common position *s* perturbed by visual noise with width *σ_V_* and the auditory perceived position will be the common position perturbed by auditory noise with width *σ_A_*. Right: Alternatively, two distinct causes may be relevant, decoupling the problem into two independent estimation problems. The causal inference model infers the probability of a causal structure with a common cause (left, *C* = 1) versus the causal structure with two independent causes (right, *C* = 2) and then derives optimal predictions from this. We introduce a single variable *C* which determines which sub-model generates the data.

We model situations in which observers are presented with simultaneous auditory and visual stimuli, and are asked to report their location(s). If the visual and the auditory stimuli have a common cause (Fig. 1, left), subjects could use the visual cue to improve the auditory estimate, and vice versa. However, in the real world we are usually surrounded by multiple sources of sensory stimulation and hence multiple sights and sounds. Therefore the nervous system cannot simply combine all signals into a joint estimate; it must infer which signals have a common cause and only integrate those. Specifically, for any pair of visual and auditory stimuli, it should also consider the alternative possibility that they are unrelated and co-occurred randomly (Fig. 1, right).

Here we developed an ideal observer that estimates the positions of cues and also whether they have a common cause. This *causal inference model* uses two pieces of information. One piece is the likelihood: the sensed visual and auditory positions, which are corrupted by noise. Because perception is corrupted by noise, any sensory stimulus does not reveal the true visual position, but rather induces a distribution of where the stimulus could be, given the stimulus. The other piece of information is the prior: from experience we may know how likely two co-occurring signals are to have a common cause versus two independent causes. The causal inference model combines those pieces of information to estimate if there is a common cause and to estimate the positions of cues (see the Methods section and Supporting Information for details, Text S1).

The causal inference model depends on four parameters characterizing the knowledge about the environment and the observer's sensory systems: the uncertainty of vision (*σ_V_*) and audition (*σ_A_*); knowledge the observer has about the spatial layout of objects, in particular how much the observer expects that objects are more likely to be located centrally (*σ_P_*, introduced to formalize that subjects have a bias to perceive stimuli straight ahead); and the prior probability that there is a single cause versus two causes (*p*_common_). These four parameters are fit to human behavior in psychophysical experiments (see Methods for details).

### Experiment 1: Auditory-visual spatial localization

Experienced ventriloquists move a puppet's mouth in synchrony with their speech patterns, creating a powerful illusion of a talking puppet. This effect is a classical demonstration of auditory-visual integration, where subjects infer that there is only a single cause (the puppet's talking) for both visual (puppet's facial movements) and auditory (speech) stimuli. Numerous experimental studies have analyzed this kind of auditory-visual cue integration and found situations in which the cues are combined and situations in which they are processed separately [2], [3], [7], [8], [10], [15], [25]–[27]. To test the causal inference model, we use a laboratory version of the ventriloquist illusion, in which brief auditory and visual stimuli are presented simultaneously with varying amounts of spatial disparity. We use the dual-report paradigm which was introduced recently to study auditory-visual numerosity judgment [13], because this provides information about the joint auditory-visual percepts of subjects.

**Figure 2:**
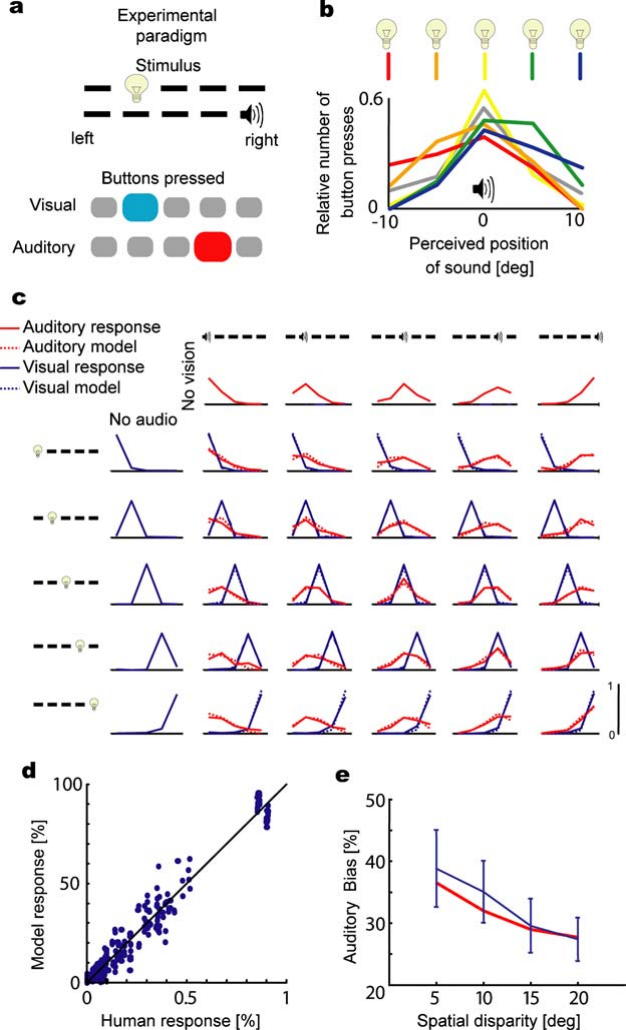
Combination of visual and auditory cues. a) The experimental paradigm is shown. In each trial a visual and an auditory stimulus is presented simultaneously and subjects report both the position of the perceived visual and the position of the perceived auditory stimuli by button presses. b) The influence of vision on the perceived position of an auditory stimulus in the center is shown. Different colors correspond to the visual stimulus at different locations (sketched in warm to cold colors from the left to the right). The unimodal auditory case is shown in gray. c) The averaged responses of the subjects (solid lines) are shown along with the predictions of the ideal observer (broken lines) for each of the 35 stimulus conditions. These plot show how often on average which button was pressed in each of the conditions. d) The model responses from c) are plotted with the human responses from c). e) The average auditory bias 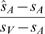, i.e. the influence of deviations of the visual position on the perceived auditory position is shown as a function of the spatial disparity (solid line) along with the model prediction (dashed line).

Nineteen subjects participated in the experiment. On each trial, subjects were presented with either a brief visual stimulus in one of five locations along azimuth, or a brief tone at one of the same five locations, or both simultaneously (see Methods for details). The task was to report the location of the visual stimulus as well as the location of the sound in each trial using two button presses (Fig. 2a).

We found that subjects show large variability across trials, shown in Fig 2b for an auditory stimulus at the central location. If subjects would not be affected by noise then we would expect a plot that has 100% of the trials as a press of the button corresponding to the central location. Instead we see a wide distribution, highlighting the presence of uncertainty in auditory perception. All modern theories of cue combination predict that two cues, presented simultaneously, will influence one another and lead to a bimodal precision that is better than the unimodal precision, as the other cue can be used to reduce uncertainty. Indeed, we found that the visual stimulus influences the estimate of the auditory stimulus when the auditory stimulus is held at a fixed location (Fig. 2b, yellow versus gray). Moreover, we find that vision does have an influence on the perception of the auditory stimulus and a visual stimulus to the left biases perception to the left (Fig 2b, red versus yellow). Subjects thus base their estimate of the auditory position on both visual and auditory cues. Moreover, subjects' estimates of the auditory position often differ from their estimates of the visual position.

To examine whether the causal inference model can account for the full range of cue combination observed in human multisensory perception, we make use of both the auditory and the visual response frequencies we measured in our experiment (Fig. 2c). Four parameters were used to fit 250 data points (25 bisensory conditions, 2 modalities, 5 buttons per modality). The causal inference model accounts for the data very well (*R* ^2^ = 0.97; *R* ^2 ^is calculated as the explained variance divided by the total variance) (Fig. 2c,d). One interesting finding is that the response distribution generally only has one peak (in Fig 2c), but its position and skewness is affected by the position of the other stimulus. The model shows this effect because it does not simply decide if there is a common cause or individual causes but considers both possibilities on each trial.

To facilitate quantitative comparison of the causal inference model with other models, we fitted the parameters individually to each subject's data using a maximum-likelihood procedure: we maximized the probability of the data under the model. For each subject, the best fit from 6 different sets of initial parameter values was used, to reduce the effect of these initial values. We did this for several different models that use previously proposed interaction priors as well as the prior derived from causal inference. We first considered two special cases of the causal inference model: pure integration (causal inference with *p*_common_ = 1) and pure segregation (causal inference with *p*_common_ = 0). We then considered two two-dimensional ad hoc priors that have been proposed in other papers. Roach et al. [18] proposed a two-dimensional (auditory-visual) prior that is defined as the sum of a Gaussian ridge along the diagonal, and a constant. This prior is somewhat similar to the causal inference prior as the constant relates to events that are independent and the Gaussian relates to sensory events that are related and thus have a common cause. Bresciani et al [14] used a special case of the Roach et al. prior ( The Shams et al. model (Shams et al, 2005) was not considered as it involves a prior specified by a large number of parameters (25)). where no constant is added to the Gaussian. According to the Bresciani prior, visual and auditory positions that are very far away from each other are extremely unlikely. According to the Roach prior, such two positions have a fixed, non-vanishing probability.

In the comparison, we obtain the predicted response distribution by integrating out the internal variables instead of equating it to the posterior distribution. This is the correct way of solving this Bayesian problem and differs from the approach taken in previous papers [13], [14], [18] (although it only affects predictions in the Roach et al. model). We measure the goodness of fit obtained from these priors relative to that obtained from the causal inference prior, using the log likelihood over the entire data set. The resulting log likelihood ratios are shown in Table 1. The causal inference model fits the data better than the other models. We also compare with an alternative model that instead of minimizing the mean squared error maximizes the probability of being correct and can exclude this model based on the presented evidence.

**Table 1:** Maximal log likelihood ratios (base *e*) across subjects of models relative to causal inference model (mean±s.e.m., see methods for details).

Model	Relative log likelihood
Causal inference	0
Causal inference with maximization	−11±3
Full integration	−311±28
Full segregation	−25±7
Roach et al.	−18±6
Bresciani et al.	−22±6

For the last two entries, we used the prior proposed by Roach et al. and Bresciani et al. together with correct inference (see text for more detail). All of the maximal likelihood ratios in the table are considered decisive evidence in favor of the causal inference prior, even when correcting for the number of parameters using the Akaike Information Criterion (AIC) or the Bayesian Information Criterion (BIC) [28]. These criteria are methods for enabling fair comparison between models. Models with more parameters always fit data better than models with fewer parameters. AIC and BIC are ways of correcting for this bias.

The parameters found in the likelihood optimization of the causal inference model are as follows. We found the visual system to be relatively precise (*σ_V_* = 2.14±0.22°) and the auditory system to be much less precise (*σ_A_* = 9.2±1.1°). We found that people have a modest prior estimating stimuli to be more likely to be central (*σ_P_* = 12.3±1.1°). Subjects have the tendency of indicating a direction that is straight ahead and the prior allows the model to show such behavior as well. The average probability of perceiving a common cause for visual and auditory stimuli is relatively low (*p*_common_ = 0.28±0.05°). This explained that the observed biases are small in comparison to the values predicted if subjects were certain that there is a common cause (Fig. 2e). In summary, the causal inference model provides precise predictions of the way people combine cues in an auditory-visual spatial localization task, and it does so better than earlier models.

In the cue combination literature, bias is commonly used as an index of crossmodal interactions. In our experiment, auditory localization bias is a measure of the influence of vision on audition and can be plotted as a function of the spatial disparity [15], [16]. Like other authors, we find that the bias decreases with increasing spatial disparity (Fig. 2e). Thus, the larger the distance between visual and auditory stimuli, the smaller is the influence of vision on audition. This result is naturally predicted by the causal inference model: larger discrepancies make the single cause model less likely as it would need to assume large noise values, which are unlikely. A model in which no combination happens at all (*p*_common_ = 0) cannot explain the observed biases (Fig. 2e) as it predicts a very small bias (*p*<0.0001 t-test). The traditional forced-fusion model [4]–[7], [29] fails to explain much of the variance in Fig. 2c (*R* ^2^ = 0.56). Moreover, this model would predict a very high bias—as vision is much more precise than audition in our experiment—and is ruled out by the bias data (Fig. 2e) (*p*<0.0001 t-test). Neither the traditional nor the no-interaction model can explain the data, whereas the causal inference model can explain the observed patterns of partial combination well (see Supporting Information, Text S1 and Fig. S2 for a comparison with some other recent models of cue combination).

### Experiment 2: Auditory-visual spatial localization with measured perception of causality

**Figure 3:**
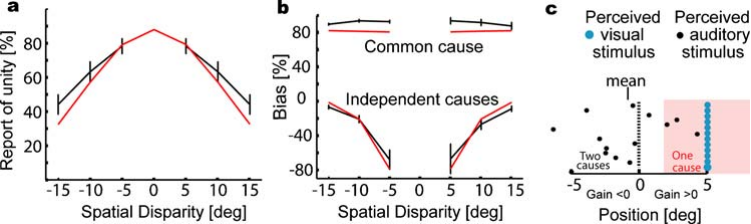
Reports of causal inference. a) The relative frequency of subjects reporting one cause (black) is shown (reprinted with permission from [15]) with the prediction of the causal inference model (red). b) The bias, i.e. the influence of vision on the perceived auditory position is shown (gray and black). The predictions of the model are shown in red. c) A schematic illustration explaining the finding of negative biases. Blue and black dots represent the perceived visual and auditory stimuli, respectively. In the pink area people perceive a common cause.

While the causal inference model accounts for the cue combination data described above, it makes a prediction that goes beyond the estimates of positions. If people infer the probability of common cause then it should be possible to ask them if they perceive a common cause versus two causes. A recent experiment asked this question [15]. We compare the predictions of the causal inference model with the reported data from this experiment. These experiments differed in a number of important respects from our experiment. Subjects were asked to report their perception of unity (i.e., whether the two stimuli have a common cause or two independent causes) on each trial. Only the location of the auditory stimulus was probed. Subjects pointed towards the location of the auditory stimulus instead of choosing a button to indicate the position (see Methods, data analysis for figure 3, for details).

The results of these experiments [15] indicate that the closer the visual stimulus is to the auditory stimulus, the more often do people perceive them as having a common cause (Fig. 3a). However, even if the two stimuli are close to one another, on some trials the noise in the perception of the auditory stimulus will sometimes lead to the perception of distinct causes. For example, a subject may hear the sound at 10° even when both stimuli really are at 0°; on such a trial, the large perceived disparity may lead the subject to report distinct causes. The model also shows the trend that with increasing disparity the probability of the perception of a common cause decreases. It explains 72% of the variance in human performance (Fig. 3a) and thus well models the human perception of causality.

We next examined how the perception of a common versus distinct causes affects the estimation of the position of auditory stimuli. The results indicate that when people perceive a common cause they point to a position that is on average very close to the position of the visual stimulus, and therefore the bias is high (Fig. 3b). If, on the other hand, subjects perceive distinct causes, they seem to not only rely on the auditory stimulus but seem to be pushed away from the visual stimulus and exhibit *negative* bias. This is a counterintuitive finding, as previous models [4]–[7], [29] predict only positive bias. Causal inference shows very similar behavior as it also exhibits negative biases, and explains 87% of the variance of the bias. The causal inference model thus accounts for the counterintuitive negative biases.

How can an optimal system exhibit negative bias? We argue that this is a selection bias stemming from restricting ourselves to trials in which causes were perceived as distinct. To clarify this, we consider, as an example, the case where the visual stimulus is 5° to the right of the center and the auditory stimulus is in the center. On some trials, the lack of precision of the auditory system will lead to the internal representation of the auditory signal being close to the visual signal and then the system infers a common cause. On those trials, the auditory stimulus will be inferred to be very close to the visual one, and the bias will be high (Fig. 3c red). On other trials, the internal representation of the auditory signal will, by chance, be far away from the visual signal, resulting in the model inferring distinct causes. The distribution of perceived auditory positions on these trials would be a truncated Gaussian distribution, with the right part of the distribution (corresponding to the perception of a common cause) cut away. This truncated distribution has a negative mean and thus leads to a negative bias. Due to this selection process, when a common cause is inferred, the perceived auditory location must be close to the visual stimulus, resulting in high bias. In contrast, when distinct causes are inferred, the perceived auditory location must be far away from the visual stimulus, and the bias thus becomes negative.

## Discussion

The causal inference model formalizes and addresses the problem that had been phrased by von Helmholtz as “probabilistically inferring the causes of cues”. This refers to the general problem that subjects are faced with: deciding which cues correspond to the same source and which are unrelated to one another. The causal inference model can predict both subjects' unity judgments and their stimulus estimates. Moreover, its interaction prior is similar to the ones that have been proposed in earlier models that did not model causal inference but only the interaction between cues [13], [14], [17], [18]. It thus provides an explanation for why models utilizing an interaction prior have been successful at modeling human performance.

In addition to providing a formalism that derives from a strong normative idea, the causal inference model leads to better fits of human performance in the auditory-visual localization task presented here. Moreover, the causal inference model can make direct predictions about the causal structure of sensory input that would have been impossible with the previous models. Inference about causal structure is an important element of the perceptual binding of multisensory stimuli: when and how do sights and sounds get paired into a unified conscious percept [22], [30]? The causal inference model presents a partial answer to this question.

As the causal inference model uses a single inference rule to account for the entire spectrum of sensory cue combination, many previous models are special cases of the model presented here, including those showing statistical optimality of complete integration (*p*_common_ = 1) when the discrepancy between the signals is small. In that case, the probability of a common cause given the sensory cues will be close to 1.

It is necessary to discuss in which way we may expect subjects to behave in an optimal way. We have shown that the assumption that subjects optimally solve causal inference problems can well predict many facets of their decision process. However, as has often been argued [31], [32], there is no reason why the nervous system should be optimal under all circumstances. For problems of high importance to everyday life, however, we should expect evolution to have found a very good solution. For this range of problems we should expect ideal observer models to make good predictions of human behavior.

This leaves the question of what neural processes underlie the causal inference computations that emerge at the behavioral level as close to Bayes-optimal. Recent work has shed some light on this issue for the case of complete integration (*p*_common_ = 1). It is well-known that neural populations naturally encode probability distributions over stimuli through Bayes' rule, a type of coding known as probabilistic population coding [33], [34]. Under the assumption of a common cause, optimal cue combination can be implemented in a biologically realistic network using this type of coding. Unexpectedly, this only requires simple linear operations on neural activity [35]. This implementation makes essential use of the structure of neural variability and leads to physiological predictions for activity in areas that combine multisensory input, such as the superior colliculus. Since complete integration is a special case of causal inference, computational mechanisms for the latter are expected to have a neural substrate that generalizes these linear operations on population activities. A neural implementation of optimal causal inference will be an important step towards a complete neural theory of multisensory perception.

In the study of higher-level cognition, many experiments have shown that people, starting from infancy, interpret events in terms of the actions of hidden causes [36]–[40]. If we see a window shatter, something or someone must have broken it; if a ball flies up into the air, something launched it. It is particularly hard to resist positing invisible common causes to explain surprising conjunctions of events, such as the sudden occurrence of several cases of the same rare cancer in a small town. These causal inferences in higher-level cognition may seem quite different than the causal inferences in sensory integration we have studied here: more deliberate, consciously accessible, and knowledge-dependent, rather than automatic, instantaneous, and universal. Yet an intriguing link is suggested by our finding. The optimal statistical principles that can explain causal inference in sensory integration are very similar to those that have recently been shown to explain more conventional hidden-cause inferences in higher-level cognition [39], [41], [42]. Problems of inferring common causes from observed conjunctions arise everywhere across perception and cognition, and the brain may have evolved similar or even common mechanisms for performing these inferences optimally, in order to build veridical models of the environment's structure.

## Materials and Methods

### Paradigm for Experiment 1

Twenty naive subjects (undergraduate students at the California Institute of Technology, ten male) participated in the experiment. All subjects were informed of the purposes of the study and gave written informed consent as approved by the local ethics committee (Caltech Committee for the Protection of Human Subjects). Subjects were seated at a viewing distance of 54 cm from a 21-inch monitor. In each trial, subjects were asked to report both the perceived visual and auditory positions using keyboard keys 1 through 5, with 1 being the leftmost location and 5 the rightmost. No feedback about the correctness of the response was given. Visual and auditory stimuli were presented independently at one of five positions. The five locations extended from 10° to the left of the fixation point to 10° to the right of the fixation point at 5° intervals, along a horizontal line 5° below the fixation point, Visual stimuli were 35 ms presentations of Gabor wavelets of high contrast extending 2° on a background of visual noise. Auditory stimuli, synchronized with the visual stimuli in the auditory-visual conditions, were presented through a pair of headphones and consisted of 35 ms white noise. Unisensory stimuli were also presented, for which there was no presentation of stimulus for the other modality. However, for the purposes of the modeling in Fig. 2c we disregarded the unimodal data because of potential attentional differences between bimodal and unimodal data. The sound stimuli were filtered through a Head-Related Transfer Function (HRTF), measured individually for each subject, using methods similar to those described by http://sound.media.mit.edu/KEMAR.html. The HRTFs were created to simulate sounds originating from the five spatial locations in the frontoparallel plane where the visual stimuli were presented. The data from one subject had to be discarded, as the fitted auditory variance of that subject was 98° and the subject was therefore effectively deaf with respect to the objective of our study. Apart from this subject, the HRTF function yielded to good auditory precision in the range of 10°.

### Generative model

A Bayesian approach based on a generative model requires one to fully specify how the variables of interest are interrelated statistically. This is done as follows. Determine if there is one cause (*C* = 1) versus two causes (*C* = 2) by drawing from a binomial distribution with *p*(*C* = 1) = *p*_common_. Outside of the experiment this will not be constant but depend on temporal delays, visual experience, context, and many other factors. In the experiments we consider all these factors are held constant so we can use a fixed *p*_common_. If there is one cause (*C* = 1), draw a position *s* from a normal prior distribution *N*(0,*σ_P_*), where *N*(*μ*,*σ_P_*) stands for a normal distribution with mean *μ* and standard deviation *σ*. It is thus more likely that a stimulus is centrally located than far to the side. We then set *s_V_ = s* and *s_A_* = *s*. If there are two causes (*C* = 2), draw positions *s_V_* and *s_A_* each independently from *N*(0,*σ_P_*). We assume that the visual and the auditory signal are corrupted by unbiased Gaussian noise of standard deviations *σ_V_* and *σ_A,_* respectively and draw *x_V_* from *N*(*s_V_*,*σ_V_*) and *x_A_* from *N*(*s_A_*,*σ_A_*). The noise is thus assumed to be independent across modalities.

### Estimating the probability of a common cause

An ideal observer is faced with the problem of inferring the causal structure, i.e., whether there is one cause or there are two causes. This inference is performed optimally using Bayes' rule: 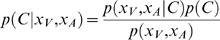
(1)Here *p*(*x_A_*, *x_V_*) must be chosen such that *p*(*C* = 1|*x_V_*, *x_A_*) and *p*(*C* = 2|*x_V_*, *x_A_*) add to 1, as we are dealing with probabilities. We thus obtain: 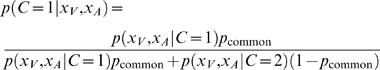
(2)For *p*(*x_V_*, *x_A_*|*C* = 1) we obtain
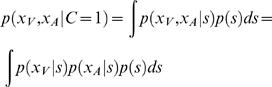
(3)All three factors in this integral are Gaussians, allowing for an analytic solution: 
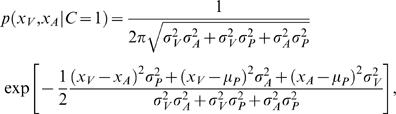
(4)where *μ_P_* = 0is the mean of the prior.

For *p*(*x_V_*, *x_A_*|*C* = 2) we note that *x*_V_ and *x_A_* are independent of each other and we thus obtain a product of two factors: 
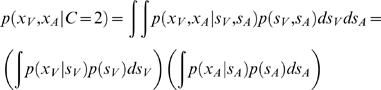
(5)Again, as all these distributions are assumed to be Gaussian, we can write down an analytic solution, 
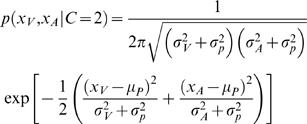
(6)For comparison with experimental data [15], we assume that the model reports a common cause when 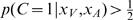. The estimation of a common cause amounts to a Bayesian model selection problem. It is mathematically similar to a mixture model for depth [24].

### Optimally estimating the position

When estimating the position of the visual target, we assume that making an error about the position of *s* in the case of a common cause is just as bad as making an error in the estimate of *s_V_*, and likewise for the position of the auditory target. We assume that the cost in this estimation process is the mean squared error [43]: 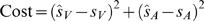
(7)An optimal estimate for the subject is the estimate that leads to the lowest expected cost under the subject's posterior belief: 
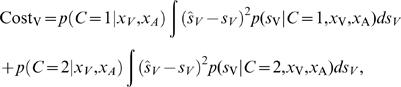
(8)where, *Ŝ_V_* is the possible estimate. In general, when the cost function is quadratic, the optimal estimation problem reduces to the problem of finding the mean of the posterior distribution. The estimate that minimizes the mean expected squared error in our case is therefore: 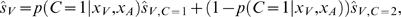
(9)and
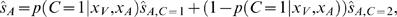
(10)where *Ŝ_C_*_ = 1_ and *Ŝ_C_*_ = 2_ are the best estimates we would obtain if we were certain about there being one or two causes, respectively. These *conditionalized* solutions are obtained by linearly weighing the different cues proportional to their inverse variances [44], as follows from the fact that the posterior is a product of Gaussians and thus a Gaussian itself. Therefore, if we know if there is a common or distinct causes we are able to analytically solve for the solution minimizing the mean squared error, which here coincides with the maximum-a-posteriori solution. The optimal visual estimate when visual and auditory sources are different is the same as when there would be only a visual signal, and likewise for the auditory estimate: 
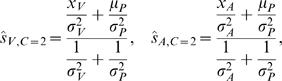
(11)When the sensory signals have a common cause, the optimal solution is: 
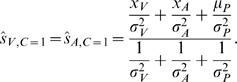
(12)While these are both linear combinations of *x_V_* and *x_A_*, the prefactors *p*(*C*|*x_V_*, *x_A_*) appearing in from Eqs. (9) and (10) are nonlinear in these two variables. Therefore, the overall optimal estimates *Ŝ_V_* and *Ŝ_A_*, when both common and independent causes are possible, are no longer linear combinations. The traditional way of studying the system by only measuring linear weights is not sufficient to fully describe a system that performs causal inference.

The predicted distribution of visual positions is obtained through marginalization: 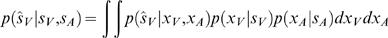
(13)These are the distributions which we can compare with experimental data, the response distributions were obtained through simulation (for more details see the Supporting Information, Text S1 and Fig. S1).

In the simulation, we ran each condition (*s_V_*, *s_A_*) 10,000 times for each subject. For each trial, we obtained estimates (*Ŝ_V_*, *Ŝ_A_*) in the way described above. To link these estimates with our data in which we have only five possible responses, we assume that people press the button which is associated with the position closest to *Ŝ*. This is equivalent to choosing the button that leads to the lowest expected mean squared error between the position indicated by the button and the position of the stimulus. This amounts to allowing only estimated values *Ŝ* that are in the set {−10°, −5°, 0°, 5°, 10°}. In this way, we obtained a histogram of responses for each subject and condition. For the model comparison in Table 1, we also considered a maximum-a-posteriori estimator, which does not use a cost function but instead selects the location from the 5-element response set that has the highest probability of being correct. To link the causal inference model with the data on causal inference [15] of figure 3 in the main text, we assume additional motor noise with width *σ*_motor_, that perturbs the estimated position *Ŝ*.

A potential problem with any kind of optimization procedure is the danger of over-fitting. To test for this with out model we split the data set in two groups, each with half the data from each subject. When optimizing on the first group, we find an excellent performance of the model (*R* ^2^ = 0.98) and when we transfer the optimized parameters to the second set of data, we still find an excellent performance (*R* ^2^ = 0.96). Overfitting is therefore not a problem with the causal inference model on this data set.

### Data Analysis for Figure 2

We choose *p*_common_, *σ_P_*, *σ_V_*, *σ_A_* to maximize the probability of the experimental data given the model. For the group analysis we obtain these 4 parameters from a dataset obtained by pooling together the data from all subjects. To obtain the histograms plotted in Figure 2C we simulate the system for each combination of cues for 10,000 times. It is a common mistake [e.g. 13], [18] to directly compare a distribution like *p*(*s_A_* | *x_V_ ,x_A_*) to the data. This is a mistake because *x_V_* and *x_A_* are internal representations that differ from trial to trial and are not accessible to the experimenter. An inferred distribution like *p*(*s_A_* | *x_V_ ,x_A_*) will give rise to a single estimate *Ŝ_V_* or *Ŝ_A_* as given by Equation 2. Only a histogram of estimates over many simulated trials can be compared with behavioral responses. This histogram will in many cases be very different in shape from *p*(*s_A_* | *x_V _, x_A_*). To fit the model to the data we maximize the probability of the data given the model using the multinomial likelihood.

### Calculating the multinomial likelihood

We need to calculate the likelihood of the data given a model and its parameters. Since the data take the form of counts of discrete outcomes (1 to 5), we use the multinomial distribution. On each trial, a response is drawn from a discrete probability distribution. Each model produces numerical approximations to the probabilities of each response in a given condition (*s_V_, s_A_* ). We will denote these *p_i_*, with *i* = 1, 2, 3, 4, 5. Suppose the observed response counts in this condition are *n_i_*, with *i* = 1, 2, 3, 4, 5. Then the probability of observing counts {*n_i_*} under this model is: 
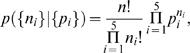
(14)where *n* is the total number of trials in this condition, 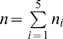, and the prefactor 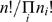 is the number of ways in which *n* responses can be split up into five counts {*n_i_*}. This gives the likelihood of the model given the data: 
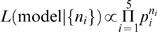
(15)Maximizing this likelihood is equivalent to maximizing its logarithm, which is given by. 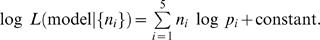
(16)


So far we have considered a single condition. Since conditions are independent of each other, the overall log likelihood is the sum of the log likelihoods in all conditions. These overall log likelihoods are compared for different models in Table 1 in the main text and are used for fitting the parameters to the causal inference model and all other models we are comparing to.

### Data analysis for Table 1

We compare how good the causal inference model is relative to other models. The way this is usually done within Bayesian statistics is by calculating the Bayes factor, the ratio of the likelihood of the data under one model to the likelihood of the data under the other model, *p*(data|model_1_)/*p*(data|model_2_). As the necessary integration of all nuisance parameters is infeasible we instead analyze the ratios of maximal probabilities which can be corrected for the different number of free variables using the BIC [45]. This ratio formalizes how much evidence the data provides for one model relative to another model and is thus a very general and mathematically precise way of quantifying the quality of a model. According to the BIC the causal inference model is preferable over the models that have fewer parameters. Table 1 reports the log maximal probability ratios for several models relative to the causal inference model.

### Data analysis for Figure 3

In these experiments, subjects report the perceived position by pointing with a laser pointer. In such cases there may be additional noise due to a misalignment of the cursor relative to the intended position of the cursor. To model this, we introduce one additional variable, motor noise. We assume that motor noise corrupts all reports, is additive and drawn from a Gaussian with width *σ*_motor_. We estimate the relevant uncertainties as follows. In both auditory and visual trials the noise will have two sources, motor noise and sensory noise. We assume that visual only trials are dominated by motor noise, stemming from motor errors and memory errors. From data presented in figure 2 of other experiments [16] where pointing responses are made in unimodal trials, we obtain *σ*_motor_ = 2.5°, and from the same graph we obtain *σ_A_* = 7.6° (because variances are added linearly). These parameters were not tuned. The other two parameters, *p*_common_ and *σ_P_*, were obtained by minimizing the squared deviation of the model predictions from the data. The choice of *p*_common_ affects the judgment of unity and thus strongly affects the bias graph as well as the commonality judgments. Large values of *p*_common_ lead to high bias and small values of *p*_common_ lead to bias values that are high only very close to zero disparity. The same parameter values are used for both graphs in figure 3.

## Supporting Information

Text S1 Supporting Information for “Causal inference in multisensory perception”
(0.11 MB DOC)

Figure S1 The interaction priors when fit to our dataset are shown for the causal inference model, the Roach et al. [1] and the Bresciani et al. priors[3].
(1.15 MB EPS)

Figure S2 The average auditory bias, i.e. the relative influence of the visual position on the perceived auditory position, is shown as a function of the absolute spatial disparity (solid line, as in Fig. 2 main text) along with the model predictions (dashed lines). Red: causal inference model. Green: behavior derived from using the Roach et al prior. Purple: behaviour derived from using the Bresciani et al prior.
(0.94 MB EPS)

## References

[1] Hatfield GC (1990). The natural and the normative theories of spatial perception from Kant to Helmholtz..

[2] Thurlow WR, Jack CE (1973). Certain determinants of the “ventriloquism effect”.. Percept Mot Skills.

[3] Warren DH, Welch RB, McCarthy TJ (1981). The role of visual-auditory “compellingness” in the ventriloquism effect: implications for transitivity among the spatial senses.. Percept Psychophys.

[4] Jacobs RA (1999). Optimal integration of texture and motion cues to depth.. Vision Res.

[5] van Beers RJ, Sittig AC, Gon JJ (1999). Integration of proprioceptive and visual position-information: An experimentally supported model.. J Neurophysiol.

[6] Ernst MO, Banks MS (2002). Humans integrate visual and haptic information in a statistically optimal fashion.. Nature.

[7] Alais D, Burr D (2004). The ventriloquist effect results from near-optimal bimodal integration.. Curr Biol.

[8] Slutsky DA, Recanzone GH (2001). Temporal and spatial dependency of the ventriloquism effect.. Neuroreport.

[9] Munhall KG, Gribble P, Sacco L, Ward M (1996). Temporal constraints on the McGurk effect.. Percept Psychophys.

[10] Jack CE, Thurlow WR (1973). Effects of degree of visual association and angle of displacement on the “ventriloquism” effect.. Percept Mot Skills.

[11] Warren DH, Cleaves WT (1971). Visual-proprioceptive interaction under large amounts of conflict.. J Exp Psychol.

[12] Gepshtein S, Burge J, Ernst MO, Banks MS (2005). The combination of vision and touch depends on spatial proximity.. J Vis.

[13] Shams L, Ma WJ, Beierholm U (2005). Sound-induced flash illusion as an optimal percept.. Neuroreport.

[14] Bresciani JP, Dammeier F, Ernst MO (2006). Vision and touch are automatically integrated for the perception of sequences of events.. J Vis.

[15] Wallace MT, Roberson GE, Hairston WD, Stein BE, Vaughan JW (2004). Unifying multisensory signals across time and space.. Exp Brain Res.

[16] Hairston WD, Wallace MT, Vaughan JW, Stein BE, Norris JL (2003). Visual localization ability influences cross-modal bias.. J Cogn Neurosci.

[17] Rowland B, Stanford T, Stein BE (2007). A Bayesian model unifies multisensory spatial localization with the physiological properties of the superior colliculus..

[18] Roach NW, Heron J, McGraw PV (2006). Resolving multisensory conflict: a strategy for balancing the costs and benefits of audio-visual integration.. Proc Biol Sci.

[19] McDonald P (2003). Demonstration by Simulation: the Philosophical Significance of Experiment in Helmholtz's Theory of Perception.. Perspectives on Science.

[20] Helmholtz HFv (1863 (1954)). On the sensations of tone as a physiological basis for the theory of music..

[21] Ernst MO (2007). Learning to integrate arbitrary signals from vision and touch.. Journal of Vision in press.

[22] Treisman A (1996). The binding problem.. Curr Opin Neurobiol.

[23] Reynolds JH, Desimone R (1999). The role of neural mechanisms of attention in solving the binding problem.. Neuron.

[24] Knill DC (2003). Mixture models and the probabilistic structure of depth cues.. Vision Res.

[25] Recanzone GH (2003). Auditory influences on visual temporal rate perception.. J Neurophysiol.

[26] Choe CS, Welch RB, Gilford RM, Juola JF (1975). The “ventriloquist effect”: visual dominance or response bias.. Perception and Psychophysics.

[27] Bertelson P, Pavani F, Ladavas E, Vroomen J, de Gelder B (2000). Ventriloquism in patients with unilateral visual neglect.. Neuropsychologia.

[28] Burnham KP, Anderson DR (2002). Model Selection and Multimodel Inference: A Practical-Theoretic Approach..

[29] Adams WJ, Graf EW, Ernst MO (2004). Experience can change the ‘light-from-above’ prior.. Nat Neurosci.

[30] Roskies AL (1999). The binding problem.. Neuron.

[31] Gould SJ, Lewontin R (1979). The Spandrels of San Marco and the Panglossian Paradigm: A Critique of the Adaptionist Programme.. Proceedings of the Royal Society B.

[32] Gigerenzer G (2001). The adaptive toolbox: toward a darwinian rationality.. Nebr Symp Motiv.

[33] Zemel RS, Dayan P, Pouget A (1998). Probabilistic interpretation of population codes.. Neural Comput.

[34] Pouget A, Dayan P, Zemel RS (2003). Inference and computation with population codes.. Annu Rev Neurosci.

[35] Ma WJ, Beck JM, Latham PE, Pouget A (2006). Bayesian inference with probabilistic population codes.. Nat Neurosci.

[36] Buehner MJ, Cheng PW, Clifford D (2003). From covariation to causation: a test of the assumption of causal power.. J Exp Psychol Learn Mem Cogn.

[37] Gopnik A, Glymour C, Sobel DM, Schulz LE, Kushnir T (2004). A theory of causal learning in children: causal maps and Bayes nets.. Psychol Rev.

[38] Saxe R, Tenenbaum JB, Carey S (2005). Secret agents: inferences about hidden causes by 10- and 12-month-old infants.. Psychol Sci.

[39] Griffiths TL, Tenenbaum JB (2005). Structure and strength in causal induction.. Cognit Psychol.

[40] Waldmann MR (2000). Competition among causes but not effects in predictive and diagnostic learning.. J Exp Psychol Learn Mem Cogn.

[41] Tenenbaum JB, Griffiths TL, Kemp C (2006). Theory-based Bayesian models of inductive learning and reasoning.. Trends Cogn Sci.

[42] Sobel D, Tenenbaum JB, Gopnik A (2004). Children's causal inferences from indirect evidence: Backwards blocking and Bayesian reasoning in preschoolers.. Cognitive Science.

[43] Kording KP, Wolpert DM (2004). The loss function of sensorimotor learning.. Proc Natl Acad Sci U S A.

[44] Ghahramani Z (1995). Computational and psychophysics of sensorimotor integration..

[45] Barnes CA (1979). Memory deficits associated with senescence: a neurophysiological and behavioral study in the rat.. J Comp Physiol Psychol.

