# Supplementary material for: Causal Inference in Multisensory Perception
Source: PLoS One. 2007 Sep 26;2(9):e943. doi: 10.1371/journal.pone.0000943 (PMC1978520; doi:10.1371/journal.pone.0000943)
Supplement: Text S1 — Supporting Information for “Causal inference in multisensory perception” (0.11 MB DOC) [file pone.0000943.s001.doc]

Causal inference in multisensory perception

Supporting Information

# Comparison with other models

In recent models of cue combination [1-3] the response distribution $p(\hat{s}_{V},\hat{s}_{A}|s_{V},s_{A})$ is treated as the posterior distribution $p(s_{V},s_{A}|x_{V},x_{A})$. This treatment leads to a significant error for non-Gaussian priors. In this paper we correctly deal with this issue by marginalizing over the latent variables $x_{A}$and $x_{V}$:

$p\left(\hat{s}_{V},\hat{s}_{A}|s_{V},s_{A}\right)=\int\int p\left(\hat{s}_{V},\hat{s}_{A}|x_{V},x_{A}\right)p\left(x_{V}|s_{V}\right)p\left(x_{A}|s_{A}\right)dx_{V}dx_{A}$.

These response distributions were obtained through simulation.

The results reported for these other models here correspond to the corrected versions of these models.

**Priors of other models**

Recently, models with so-called interaction priors have been proposed [1-3], which define explicit interactions between the cues. These models do not assume full integration and thus lead to much better predictions than the traditional forced-fusion model. While the model by Shams et al. [2] was the first of such models, we will not make a comparison with that model here as it has many more free parameters than the causal inference model (and in fact it may be seen as a superset of all the models tested here). Table 1 in the main text analyses how well each of the models predicts the data.

Given an interaction prior $p\left(s_{A},s_{V}\right)$, we obtain the bisensory posterior distribution through Bayes’ rule,

$p\left(s_{A},s_{V}|x_{A},x_{V}\right)\propto p\left(s_{A},s_{V}\right)p\left(x_{A}|s_{A}\right)p\left(x_{V}|s_{V}\right)$.

In earlier models (Bresciani et al.; Roach et al.), the interaction prior is assumed to take a specific form. In Bresciani et al., it is a Gaussian ridge on the diagonal with width $\sigma_{\text{coupling}}$:

$$p\left(s_{A},s_{V}\right)\propto e^{-\frac{\left(s_{A}-s_{V}\right)^{2}}{2\sigma_{\text{coupling}}^{2}}}$$

A straightforward computation then allows us to obtain the auditory posterior:

$$\begin{matrix} p\left(s_{A}|x_{A},x_{V}\right)\propto\int p\left(s_{A},s_{V}\right)p\left(x_{A}|s_{A}\right)p\left(x_{V}|s_{V}\right)ds_{V} \\ \propto\int e^{-\frac{\left(s_{A}-s_{V}\right)^{2}}{2\sigma_{\text{coupling}}^{2}}}p\left(x_{A}|s_{A}\right)p\left(x_{V}|s_{V}\right)ds_{V} \\ \propto p\left(x_{A}|s_{A}\right)\int N\left(s_{V};s_{A},\sigma_{\text{coupling}}\right)N\left(s_{V};x_{V},\sigma_{V}\right)ds_{V} \\ =N\left(s_{A};x_{A},\sigma_{A}\right)N\left(s_{A};x_{V},\sqrt{\sigma_{\text{coupling}}^{2}+\sigma_{V}^{2}}\right) \\ \propto N\left(s_{A};\frac{x_{A}\sigma_{A}^{-2}+x_{V}\left(\sigma_{\text{coupling}}^{2}+\sigma_{V}^{2}\right)^{-1}}{\sigma_{A}^{-2}+\left(\sigma_{\text{coupling}}^{2}+\sigma_{V}^{2}\right)^{-1}},\frac{1}{\sqrt{\sigma_{A}^{-2}+\left(\sigma_{\text{coupling}}^{2}+\sigma_{V}^{2}\right)^{-1}}}\right) \end{matrix}$$

where $N\left(x;\mu,\sigma\right)$ is the value of the normal distribution with mean $\mu$ and standard deviation $\sigma$ evaluated at *x*. Because the posterior distribution is a pure Gaussian, it can be identified with the response distribution. The expression for the visual distribution is obtained by interchanging *V* and *A*.

In the work by Roach et al. [1], a more general form of interaction prior is used, consisting of the same Gaussian ridge on the diagonal, but added to that a constant background $\omega$:

$$p\left(s_{A},s_{V}\right)\propto\omega+e^{-\frac{\left(s_{A}-s_{V}\right)^{2}}{2\sigma_{\text{coupling}}^{2}}}$$

This gives rise to the following posterior distribution:

$\begin{matrix} p\left(s_{A}|x_{A},x_{V}\right)\propto\int\left(\omega+e^{-\frac{\left(s_{A}-s_{V}\right)^{2}}{2\sigma_{\text{coupling}}^{2}}}\right)p\left(x_{A}|s_{A}\right)p\left(x_{V}|s_{V}\right)ds_{V} \\ =\omega p\left(x_{A}|s_{A}\right)\int p\left(x_{V}|s_{V}\right)ds_{V}+p\left(x_{A}|s_{A}\right)\sqrt{2\pi\sigma_{\text{coupling}}^{2}}\int N\left(s_{V};s_{A},\sigma_{\text{coupling}}\right)N\left(s_{V};x_{V},\sigma_{V}\right)ds_{V} \\ =\omega N\left(s_{A};x_{A},\sigma_{A}\right)+\sqrt{2\pi\sigma_{\text{coupling}}^{2}}N\left(s_{A};x_{A},\sigma_{A}\right)N\left(s_{A};x_{V},\sqrt{\sigma_{\text{coupling}}^{2}+\sigma_{V}^{2}}\right) \\ =\omega N\left(s_{A};x_{A},\sigma_{A}\right)+ \\ +\sqrt{2\pi\sigma_{\text{coupling}}^{2}}N\left(x_{A};x_{V},\sqrt{\sigma_{\text{coupling}}^{2}+\sigma_{V}^{2}+\sigma_{A}^{2}}\right)N\left(s_{A};\frac{x_{A}\sigma_{A}^{-2}+x_{V}\left(\sigma_{\text{coupling}}^{2}+\sigma_{V}^{2}\right)^{-1}}{\sigma_{A}^{-2}+\left(\sigma_{\text{coupling}}^{2}+\sigma_{V}^{2}\right)^{-1}},\frac{1}{\sqrt{\sigma_{A}^{-2}+\left(\sigma_{\text{coupling}}^{2}+\sigma_{V}^{2}\right)^{-1}}}\right) \end{matrix}$ The best estimate under is the mean of this distribution, which is

$$\hat{s}_{A}=\frac{\omega x_{A}+\sqrt{2\pi\sigma_{\text{coupling}}^{2}}N\left(x_{A};x_{V},\sqrt{\sigma_{\text{coupling}}^{2}+\sigma_{V}^{2}+\sigma_{A}^{2}}\right)\frac{x_{A}\sigma_{A}^{-2}+x_{V}\left(\sigma_{\text{coupling}}^{2}+\sigma_{V}^{2}\right)^{-1}}{\sigma_{A}^{-2}+\left(\sigma_{\text{coupling}}^{2}+\sigma_{V}^{2}\right)^{-1}}}{\omega+\sqrt{2\pi\sigma_{\text{coupling}}^{2}}N\left(x_{A};x_{V},\sqrt{\sigma_{\text{coupling}}^{2}+\sigma_{V}^{2}+\sigma_{A}^{2}}\right)}$$

The expression for the visual estimate is obtained by interchanging *V* and *A*. This model is more similar to the causal inference model, because the estimates are nonlinear functions of $x_{V}$ and $x_{A}$. Because of that reason as well, the response distribution can now no longer be identified with the response distribution.

There is a direct link between the causal inference model and these models with interaction priors. The causal inference model can be recast as a model with an interaction prior by integrating out the latent variable *C*:

$p\left(s_{V},s_{A}\right)=p_{co\text{mmon}}\delta\left(s_{V}-s_{A}\right)p\left(s_{A}\right)+\left(1-p_{\text{common}}\right)p\left(s_{V}\right)p\left(s_{A}\right)$.

All predictions about position estimates with this model are retained. However, this model no longer explicitly represents whether there is a single cause or alternatively two independent causes. This explains why those models are relatively successful at explaining the data.

Fig 1S shows the priors obtained from the models discussed here, after fitting their parameters to optimally describe the data.

**Bias as a function of disparity**

In the main text we have used the probability of the data given the model as a measure for the quality of each model. We found that the causal inference model best explains the data. Although such inferential statistics are a good tool to compare models, we are also interested in the question why the causal inference model performs better. To get an understanding of the differences we turn to the graph showing bias as a function of disparity (Fig. 2S). The data shows that the bias decreases with increasing spatial disparity. The further the distance between visual and auditory stimuli, the smaller is the influence of vision on audition. This result is naturally predicted by the causal inference model; larger discrepancies make the single cause model less likely as it needs to assume large noise values that are unlikely. The joint prior used by Bresciani et al. [3] predicts a bias that is largely invariant to the disparity. However, lacking a way to represent that two cues may be entirely independent, it underestimates the derivative of the bias graph.

**References**

1. Roach NW, Heron J, McGraw PV (2006) Resolving multisensory conflict: a strategy for balancing the costs and benefits of audio-visual integration. Proc Biol Sci 273: 2159-2168.

2. Shams L, Ma WJ, Beierholm U (2005) Sound-induced flash illusion as an optimal percept. Neuroreport 16: 1923-1927.

3. Bresciani JP, Dammeier F, Ernst MO (2006) Vision and touch are automatically integrated for the perception of sequences of events. J Vis 6: 554-564.

**Figure Legends:**

**Figure 1, supporting information:** The interaction priors when fit to our dataset are shown for the causal inference model, the Roach et al. [1] and the Bresciani et al. priors[3].

**Figure 2, supporting information:** The average auditory bias$\frac{\hat{s}_{A}-s_{A}}{s_{V}-s_{A}}$, i.e. the relative influence of the visual position on the perceived auditory position, is shown as a function of the absolute spatial disparity (solid line, as in Fig. 2 main text) along with the model predictions (dashed lines). Red: causal inference model. Green: behavior derived from using the Roach et al prior. Purple: behaviour derived from using the Bresciani et al prior.
